# 

**Published:** 1900-08-18

**Authors:** 


### NOTICE TO CORRESPONDENTS.

All MS., Letters, Books for Review, and other matters intended for the Editor should be addressed The Editor, "The Hospital," The Hospital Building, 28 & 29, Southampton Street, Strand, W.C.

The Editor cannot undertake to return rejected MS., even when accompanied by stamped directed envelope.

### Notice to Advertisers and Subscribers.

All Advertisements, Orders for Copies of The Hospital, and Business Communications should be addressed to **THE MANAGER (Not to the Editor)**, The Hospital, The Hospital Building, 28 & 29, Southampton Street, Strand, London, W.C.

The Cost of Subscription, post free, is as follows:—Per week, 2½d.; per quarter, 2s. 8d.; per half-year, 5s. 3d.; per annum, 10s. 6d. Foreign:—Per annum, 15s. 2d., payable, in all cases, in advance.

**NOTICE.—Vol. XXVII. of "The Hospital" (October, 1899, to April, 1900), in handsome cloth binding, is now on sale at the Office of this Journal, price 7s. 6d. Cases for binding the Numbers contained in this Volume 1s. 6d. Index to Vol. XXVII., 2½d., post free.**

**The Monthly Part for August is now ready. Price 6d., by post 8d.**

### SCRAPS AND GLEANINGS.

The annual meeting of the National Association for Promoting the Welfare of the Feeble Minded was recently held at Aubrey House, Kensington, by kind permission of Mrs. Alexander. A large company was present, and the meeting passed off satisfactorily.

Mr. W. Nocton, J.P., has placed at the disposal of officers wounded in the South African War a bungalow hospital which has just been built in his beautiful park at Langham Hall, near Colchester, and the opening ceremony was performed by General Sir William Gatacre.

A new convalescent home for children has just been opened at Hayling Island, having been built and presented to the Ministering Children's League by the Countess of Meath. The home, which will accommodate 24 children, is built on the eastern shore, about four miles from the railway station.

Messrs. Scrubb and Co. have received an unsolicited letter of praise from the Executive Committee of the American Hospital Ship for South Africa testifying to the great use of the cloudy fluid ammonia sent by them to the hospital ship "Maine." They also sent their antiseptic skin soap, which was found of high quality and general excellence.

The Glasgow Parish Council have accepted plans for the erection of the new hospital at Stobhill, the estimated cost being £163,431. The hospital is to consist of 14 wards, with accommodation in each for 26 patients, and this will give an allowance of 12,000 cubic feet per patient. A railway siding, estimated to cost £1,000, will connect the institution with the Caledonian Railway, thus enabling stores to be discharged direct.

At the second annual meeting in connection with the Melbourne Hall (Leicester) Convalescent Home the Rev. W. Y. Fullerton, in presenting the report, stated that subscriptions had been contributed of upwards of £90, and a cheque for £100 for furnishing the hall had been sent anonymously. Reference was made to the matron, Mrs. Lawton, who had been spoken of by several visitors as the "guardian angel" of the home, and a vote of thanks was passed to her and to Mrs. Hardy-Smith.

A hospital for the treatment of tropical diseases is to be erected in Liverpool in memory of the late Miss Mary Kingsley. Already one subscription for £1,000 and two for £500 each have been received, and several others have been promised.

The annual report of the Liverpool Convalescent Institution states that during the year 1899 the number of patients (although six less than that of the previous year) was 25 per cent. above the average for the past 10 years. The annual subscriptions, which amounted to £944 odd, showed an increase of £46 compared with the preceding year. The accounts showed an increase in the debit balance from £63 to £354, this being due in great measure to certain exceptional but necessary outlay; and Mr. Joseph Wilson offered to subscribe £50 towards clearing off this balance.

At the meeting of the Forfar District Committee recently held at Forfar the subject of the erection of the Infectious Diseases Hospital near Forfar was again discussed. After the Clerk had read a report of a visit of inspection of the Sub-committee, in which it was stated that they were quite satisfied with the manner in which the work of erection was progressing, and of the quality of the material being used, Mr. D. M. Graham (at whose instigation the inspection had been made) again stated most emphatically that he did not consider the building was being erected in accordance with the specification, and that he repudiated everything connected with it. The subject was in the result allowed to drop.

### THE STUDENT OF MEDICINE.

### APPOINTMENTS.

C. M. Atkinson, M.R.C.S., L.R.C.P.Lond., appointed Medical Officer of the East Ashford Union Workhouse. F. S. Beachcroft, M.R.C.S., L.R.C.P.Lond., appointed Medical Officer for the Second District and Workhouse of the Petworth Union. F. C. Blakiston, M.R.C.S., L.R.C.P.Lond., appointed Junior Assistant Medical Officer to the Wandsworth and Clapham Union Infirmary. G. H. Brown, L.R.C.P.Edin., L.F.P.S.Glasg., appointed Medical Officer for the Nantyglo and Blaina District of the Bedwelty Union. C. Campbell, L.R.C.P.I., M.R.C.S.Eng., appointed Certifying Factory Surgeon for the Saddleworth District of the West Riding of Yorkshire. L. Cooke, L.R.C.P., L.R.C.S.Edin., appointed Medical Officer for the Aspull District of the Wigan Union. W. Cowie, M.A., M.B., C.M.Aberd., appointed Medical Officer for the Charlton District of the Woolwich Union. A. K. Crossfield, L.R.C.P., L.R.C.S.Edin., appointed Medical Officer for the Dartmouth District of the Totnes Union. R. S. Ellis, M.B., Ch.B.Edin., appointed Assistant Resident Medical Officer to the St. Mary's Children's Hospital, Plaistow. F. T. Gourlay, M.B., C.M.Edin., appointed Medical Officer for the Shiffnal and Stockton Districts and the Workhouse of the Shiffnal Union. L. Grant, M.D.Edin., appointed Medical Officer to the Ballachulish Slate Quarries. G. C. Hall, M.R.C.S.Eng., appointed Medical Officer for the Islip District of the Bicester Union. J. P. Henry, B.A., M.D., B.Ch., appointed Honorary Ophthalmic Surgeon to the Italian Hospital, Queen Square. A. M. Martin, M.B., B.S.Durh., appointed Honorary Surgeon to the Royal Infirmary, Newcastle-upon-Tyne. C. W. H. Newington, M.R.C.S., L.R.C.P., L.S.A., appointed Medical Officer and Public Vaccinator of the Sixth District of the Sevenoaks Union. R. J. Pritchard, M.R.C.S., L.R.C.P.Lond., appointed Medical Officer for the Third District of the Petworth Union. F. A. Southam, F.R.C.S.Eng., appointed Professor of Clinical Surgery at Owens College, Manchester. J. W. Unsworth, M.B., Ch.B.Vict., appointed Medical Officer for the Blackrod District of the Wigan Union. D. Wield, M.D., M.A.Edin., appointed Medical Superintendent to the "Contact Area," Brisbane, in connection with the outbreak of bubonic plague in Brisbane. E. V. R. Woodford, M.R.C.S., L.R.C.P.-Lond., appointed Medical Officer for the First District and the Workhouse of the Abingdon Union. G. A. Wright, F.R.C.S.-Eng., appointed Professor of Systematic Surgery at Owens College, Manchester.

### THE NURSE'S REPORT BOOK.

Foolscap 4to., Black cover, **6d.** net, post free; or 12 for **4s. 6d.**, post free.

**Compiled by K. H. (Cert.)**

H. J. GLAISHER, 57, Wigmore Street, Cavendish Square, W.

